# The relationship of cerb B 2 expression with estrogen receptor and progesterone receptor and prognostic parameters in endometrial carcinomas

**DOI:** 10.1186/1746-1596-5-13

**Published:** 2010-02-18

**Authors:** Aylin Ege Gul, Sevinc Hallac Keser, Nagehan Ozdemir Barisik, Nilufer Onak Kandemir, Caglar Cakır, Sibel Sensu, Nimet Karadayi

**Affiliations:** 1 Pathology Department, Dr. Lütfi Kırdar Kartal Educational and Research Hospital, Istanbul, Turkey; 2 Pathology Department, Karaelmas University, Faculty of Medicine, Zonguldak, Turkey

## Abstract

**Background:**

Endometrial carcinoma (EC) is the most common malignancy of the female genital tract. Gene alterations and overexpression of various oncogenes are important in tumor development. The human HER 2 neu (c-erbB-2) gene product is a transmembrane receptor with an intracellular tyrosine kinase that plays an important role in coordinating the endometrial growth factor receptor signaling network. The aim of this study was to investigate the expression of c-erbB-2 in endometrial cancer, to study its correlation to established prognostic parameters and estrogen receptor (ER) and progesterone receptor (PR) status.

**Methods:**

Immunohistochemical (IHC) analyses of ER, PR and c-erbB-2 were performed in 72 EC cases.

**Results:**

We detected a positive staining with c erbB 2 in 18.1% of the cases and determined a statistically significant relation between c-erbB-2 and PR. We could not find a statistically significant relation between c-erbB-2 staining and ER. There was not a statistically significant difference between c-erbB-2 and histological grade. The highest level of c-erbB-2 was found in grade 2 cases. There was not any statistically significant relation between c-erbB-2 and menstrual status, myometrial invasion, lymph node status, stage and survival.

**Conclusions:**

Although our study provides additional evidence of the potential prognostic role of c-erbB-2, further prospective and controlled studies are required to validate their clinical usefulness.

## Background

EC is the most common malignancy of the female genital tract in industrialized countries, and occurs predominantly after menopause [1-14].

Histological grade is strongly associated with prognosis, stage, lymph-node metastasis and myometrial invasion. Nonendometrioid carcinomas are considered high-grade tumors and thus need not be graded. Grade is one of the prognostic factors applied in clinical decisions regarding treatment. The most frequently used grading criteria are the ones of International Federation of Gynecology and Obstetrics (FIGO) and the World Health Organization (WHO), which include both architectural and nuclear features [1]. The presence of metastases in the lymphatic or vascular spaces of the uterus and lymphovascular space involvement is an important prognostic factor for relapse of disease and poor survival, and it is independent of histological grade or depth of myometrial invasion [1].

Steroid hormones, especially estrogen, play an important role in the pathogenesis of EC. It is postulated that estrogen exposure unopposed by progestins increases the risk of endometrial hyperplasia and cancer [1,9,11,15]. Estrogen receptors (ERs) and progesterone receptors (PRs) are generally decreased in EC compared with endometrial hyperplasia and the loss of receptors is a part of the carcinogenesis of the endometrium [9]. In recent years, the molecular analysis of EC has identified abnormalities in the expression, structure, or activity of oncogene products which can contribute to the development and maintenance of the malignant phenotype [3].

HER-2, also known as c-erbB-2, is a 185 kd transmembrane receptor protein encoded by HER-2/neu gene, which is localized on chromosome 17. HER-2 is a member of the epidermal growth factor receptor family with tyrosine kinase activity together with HER-1, HER-3, and HER-4. Epidermal growth factor (EGF) receptor related with growth factors has a regulatory role, particularly by influencing the mitogenic activity [2,4-7,12,13].

When c-erbB-2 is normally expressed, it leads to the combination of a few copies of c-erbB-2 heterodimers and the c-erbB-2-mediated signaling is weak, resulting in a normal cell growth [2]. Overexpression of c-erbB-2 has been associated with a more agressive biological behavior of human tumors including breast and ovarian cancer, prostate, bladder, cervical cancer, and EC [2,5,6]. Overexpression of the c-erbB-2 oncogene occurs in about 10% to 40% of EC and has been associated with other adverse prognostic factors, including advanced stage, higher grade and worsened overall survival [1,7,16-20].

The aim of this study was to investigate the expression of c-erbB-2 in endometrial cancer, to study its correlation to established prognostic parameters and estrogen receptor (ER) and progesterone receptor (PR) status.

## Methods

The study was performed on 72 endometrial adenocarcinoma cases diagnosed between 2004-2007 at Dr. Lutfi Kirdar Training and Research Hospital Pathology Clinic. The patients were allocated to two groups according to their menstrual status as premenopausal and postmenopausal. All the cases were hysterectomy specimens and were diagnosed as endometrioid EC. Histological characteristics were abstracted from the original pathology reports. The hematoxylin-eosin-stained sections of the hysterectomy specimens from all the patients were re-evaluated by two of the authors (AEG, SHK) to confirm the histologic type, grade, depth of myometrial invasion and stage. The histological classification which was in consistency with the classification of the WHO architectural grading was based on the degree of glandular differentiation in accordance with the FIGO guidelines. The cases were categorized as well-differentiated grade 1 (G1), moderately differentiated grade 2 (G2), and poorly differentiated grade 3 (G3) adenocarcinomas according to the WHO criteria. Depth of myometrial invasion was categorized as no myometrial invasion, invasion within 1/2 thickness of the myometrium, and invasion of >1/2 thickness of the myometrium. The cases of which lymph node dissection was performed were classified into two groups; with lymph node metastasis and without lymph node metastasis. Staging was performed according to the TNM and FIGO classification. FIGO stage was ascertained from the surgical pathology reports. The paraffin block that best represented the tumor was selected for the immunohistochemical examination. To collect the necessary information about the patient's survival we tried to reach all of the cases and we were able to contact 44 patients by phone through the information available in the oncology clinic records, but we could not contact 28 patients.

### Immunohistochemistry

Three μm sections obtained from the selected paraffin blocks were taken on to adhesive-coated slides and kept overnight in 37°C incubator. After deparaffinization, the slides were passed from graded alcohols for 15 min and rinsed in distilled water. The slides were then placed in pH 6.0 citrate buffer for antigen retrieval process in microwave oven for 20 min. Endogenous peroxidase activity was blocked by 10 min. of incubation with 3% hydrogen peroxide. To prevent nonspecific bindings, blockage was done for 10 min. ([Dako], Cytomation protein block serum-free, ref x0909, lot 10016138). The primary antibodies used were as follows: polyclonal rabbit antihuman c-erbB-2 oncoprotein ([Dako] Cytomation EnVision A0485 affinity isolated/10 dilution 90 min.) monoclonal rabbit ER antibody (rabbit monoclonal antihuman) ([Spring Bioscience], Code M3011, Lot. 70918, 1: 100 dilution, 90 min.), monoclonal mouse antihuman PR antibody ([Dako] Cytomation EnVision Clone PgR636, Lot. 00009021, 1:100 dilution 90 min.). Secondary antibodies were used for 30 min. with primary antibody enhancer ([Spring Bioscience] cat: DPE-125, Lot:70828) for 30 min. The slides were incubated for 30 min. with polyvalent HRP polymer ([Spring Bioscience] cat: DPE-125, Lot:70918). AEC chromogen system was applied to slides for 15 min ([Spring Bioscience] catalog: ASS-125 ready to use). Contrast staining was done with Mayer's hematoxylene and slides were finally covered with water-based cover material. For c-erbB-2, ER and PR, breast tissue, positivity of which had been proven previously was used as a positive control. Immunoreactivity of c-erbB-2 was observed in the cell membrane and was scored semiquantitatively using the Food and Drug Administration (FDA) approved scoring system as: 0, no immunostaining; 1+, incomplete membranous immunostaining of <10% of tumor cells; 2+, weak complete membranous immunostaining of >10% of tumor cells; 3+, strong complete membranous staining of >10% of tumor cells. Scores of 0 or 1+ indicated a negative result, while scores of 2+ and 3+ were regarded as positive c-erbB-2 expression [7]. ER and PR examinations were done based on the percentage of the stained cells and the intensity of the nuclear staining. The percentage of positive cells was graded as follows: 1 = 0 to 25% of the nuclei stained; 2 = 26 to 75% of nuclei stained, 3 = more than 76% of the nuclei stained. The staining intensity was scored as follows: 1 = absent or weak, 2 = strong, 3 = very strong. The sum of both of the parameters determined the IHC score. Tumors were classified to three categories depending on the IHC score. Category I corresponded to a score of 2 and category II to a score of 3 or 4, and category III to a score of 5 or 6. Category-I tumors were considered as immunonegative, whereas category II and III tumors were considered as immunopositive [21]. The relationships between c-erbB-2, ER-PR status, menstrual status, histological grade, myometrial invasion depth, lymph node status, and stage were investigated.

### Statistical Analysis

SPSS (Statistical Package for Social Sciences) for Windows 15.0 software was used for the statistical analysis. For the comparison of quantitative data, Chi-square and crosstabulation tests were used. Results were analysed at p < 0.05 significance and 95% confidence interval.

## Results

The present study included 72 patients who had undergone surgery due to EC of the uterine corpus. Twenty two patients (30.6%) were premenopausal, and 50 patients (69.4%) were postmenopausal. The mean age of the patients was 58.3 years (range, 30-81). When classified according to grades, 25 patients (34.7%) were G1, 39 patients were (54.2%) G2, and 8 patients were (11.1%) G3. Invasion depth was less than 1/2 in 41 (56.9%) patients, and more than 1/2 in 25 (34.7%) patients. However, in 6 (8.3%) patients myometrial invasion was not observed and tumors were limited to the endometrium. There were 34 cases that lymph node dissection was done; in 4 (11.8%) cases lymph node metastases were detected. There were 28 cases at stage I and there was only one case at stage II. There were 5 cases at stage III. We were able to collect the information of 49 patients; 44 of them survived but 5 of them died. The follow-up period was at least 24 months. The characteristics of the cases are given in detail on Table 1.

**Table 1:** Distribution of parameters

		n	*%*
**Menstrual Status**	**Premenopausal**	22	*30,6*
	**Postmenopausal**	50	*69,4*

**Histological grade**	**Grade 1**	25	*34,7*
	**Grade 2**	39	*54,2*
	**Grade 3**	8	*11,1*

**Invasion depth**	**<1/2**	41	*56,9*
	**>1/2**	25	*34,7*
	**Limited to endometrium**	6	*8,3*

**Lymph-Node Status**	**Missing (no lymph node)**	38	*52,8*
	**Positive**	4	*5,6*
	**Negative**	30	*41,7*

	**I**	28	*82,4*
**Stage**	**II**	1	*2,9*
	**III**	5	*14,7*

**c-erbB-2**	**Positive**	13	*18,1*
	**Negative**	59	*81,9*

**Estrogen**	**Positive**	61	*84,7*
	**Negative**	11	*15,3*

**Progesterone**	**Positive**	59	*81,9*
	**Negative**	13	*18,1*

**Survival**	**Alive**	44	*89,7*
	**Death**	5	*10,3*

**Figure 1:**
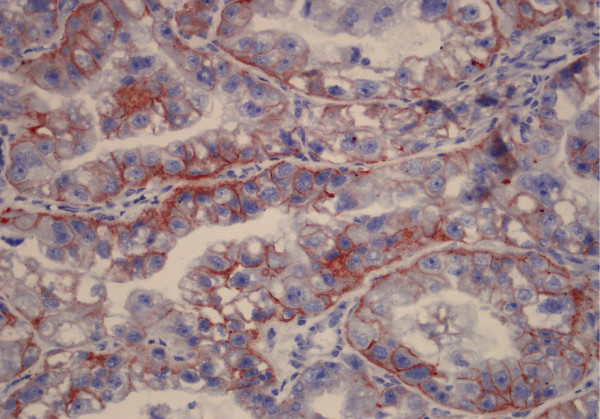
**Positive reaction for c-erbB-2 in endometrial adenocarcinoma (IHC×400)**.

Among all 18.1% had positive staining with c-erbB-2 (Figure 1). C-erb-B2 was positive in 6 (27.3%) of the 22 premenopausal patients, and also positive in 7(14%) of the 50 postmenopausal patients. C-erbB-2 positivity was determined in 3 cases that were G1 (12%), 9 cases that were G2 (23.1%), and one case that was G3 (12.5%). No significant difference was determined (p > 0,05) between the histological grades and the cases that had c-erbB-2 expression. The highest c-erbB-2 positivity was in grade 2 cases (23.1%). There were ten (24.4%) myometrial invasion cases less than 1/2. Two (8%) myometrial invasion cases which were more than 1/2 and 1 (16.7%) case with no myometrial invasion had a positive reaction with c-erbB-2. There was no statistically significant difference between the invasion depth and the cases with c-erbB-2 expression. One case (25%) with lymph node metastasis showed positive staining with c-erbB-2. Positive staining with c-erbB-2 was also detected in 2 cases (6.7%) that lymph node metastases was not detected. Immunoreactivity with c-erbB-2 was detected in 2 cases in stage I (7.1%), there was only one case detected in stage II and no staining with c-erbB-2 was observed in this case. Immunoreactivity with c-erbB-2 was detected in one case (20%) in stage III. The proportion of the survived patients that were staining with cerb-B-2 was (18.18%). There was not a statistically significant relation with survival and cerb-B-2.

**Figure 2:**
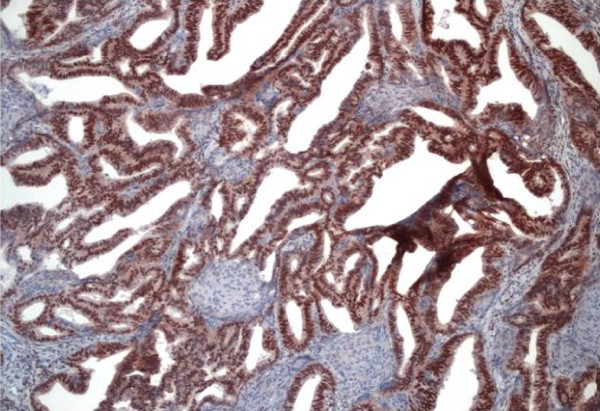
**Strong immunoreactivity for PR in the endometrial adenocarcinoma (IHC×200)**.

ER positivity was determined in 61 of the 72 cases (84.7%) and PR positivity was determined in 59 of the 72 cases (81.9%) (Figure 2). The proportion of the positive staining between ER and grades (1-2-3) were 24 (96%), 33(84.6%) and 4(50%), respectively. The proportion of the staining was observed but there was not a statistically significant relation between ER and the grades. PR expression was determined relatively high in proportion among the G1 and G2 cases. In G3 cases PR expression ratio was low and it was statistically significant (p < 0.05). Positive staining was observed both in ER and PR in 23 (82.14%) out of the 28 stage I cases. There was only one case in stage II and positive staining was observed both in ER and PR. All of the 5 cases in stage III showed positive staining with ER but only 3 of the cases had positive staining with PR. There was not any statistically significant relations between ER-stage-survival and PR-stage-survival. Nine cases (14.8%) of all with ER positivity and 8 (13.6%) of the cases with PR positivity showed a positive staining with c-erbB-2 staining characteristics are summarized in Table 2, 3 and 4.

**Table 2:** c-erbB-2 score evaluations

		c-erbB-2 score		
			
		Negative n (%)	Positive n (%)	*P†*
**Menstrual Status**	**Premenopausal**	16 (72,7)	6 (27,3)	
	**Postmenopausal**	43 (86)	7 (14)	***0,177***

**Histological Grade**	**Grade 1**	22 (88)	3 (12)	***0,484***
	**Grade 2**	30 (76,9)	9 (23,1)	
	**Grade 3**	7 (87,5)	1 (12,5)	

**Invasion Depth**	**<1/2**	31 (75,6)	10 (24,4)	***0,243***
	**>1/2**	23 (92)	2 (8)	
	**Limited to endometrium**	5 (83,3)	1 (16,7)	

**Lymph Node Status**	**Positive**	3 (75)	1 (25)	***0,615***
	**Negative**	28 (93,3)	2 (6,7)	

**Stage**	**I**	26 (92,9)	2 (7,1)	
	**II**	1 (100)	0 (0)	
	**III**	4 (80)	1 (20)	

**Estrogen**	**Positive**	52 (85,2)	9 (14,8)	***0,086***
	**Negative**	7 (63,6)	4 (36,4)	

**Progesterone**	**Positive**	51 (86,4)	8 (13,6)	***0,035***
	**Negative**	8 (61,5)	5 (38,5)	

**Survival**	**Alive**	36 (81,82)	8 (18,18)	***0,215***
	**Death**	5 (100)	0 (0)	

†: Chi square test. p < 0,05 significant; p > 0,005 insignificant

**Table 3:** The Relationship Between Estrogen with Grade, Stage, Survival

		Estrogen		
			
		Negative (n = 11) n(%)	Positive(n = 61) n(%)	*P*
**Survival**	**1 (**alive)	3(6,8)	41 (93,2)	**0,592**
	**2 (**death)	1(20,0)	4 (80,0)	

**Grade**	**1**	1 (9,1)	25 (40,3)	0,006**
	**2**	6 (54,5)	33 (53,2)	
	**3**	4 (36,4)	4 (6,5)	
	
				

**Stage**	**I**	I5(17,9)	23(82,1)	**0,543**
	**II**	0 (0)	1(100)	
	**III**	0 (0)	5 (100)	

**Statistically insignificant: p value > 0,005

*Statistically significant p value < 0,05

**Table 4:** The Relationship Between Progesterone with Grade, Stage, Survival

		Progesterone		
			
		Negative (n = 13) n(%)	Positive (n = 59) n(%)	*p*
**Grade**	**1**	2 (17,8)	23 (82,2)	0,002**
	**2**	6 (15,4)	33 (84,6)	
	**3**	5 (62,5)	3 (37,5)	

**Stage**	**I**	4 (14,8)	23 (85,2)	**0,085**
	**II**	0 (0)	1 (100)	
	**III**	2 (40)	3 (60)	

**Survival**	**1 (alive)**	5 (11,4)	39 (88,6)	**0,076**
	**2 (death)**	2 (40)	3 (60)	

**Statistically insignificant: p value > 0,005

*Statistically significant p value < 0,05

There was no statistically significant relation between c-erbB-2 and ER (p > 0.05). There was a statistically significant relation between c-erbB-2 and PR (p < 0.05). C-erbB-2 expression was observed in a higher ratio in PR negative cases compared with PR positive cases.

## Discussion

C-erbB-2 protein overexpression and c-erbB-2 gene amplification are frequently associated with a more aggresive and chemoresistant disease in cancer patients [2]. C-erbB-2 is reported in 9-60% of the ECs [17]. According to the results reported by Brys et al. the erbB-1 and erbB-2 mRNA expression level was significantly higher in malignant human endometrium compared to benign tissue. Moreover, a significant correlation between erbB-1 overexpression and tumor differentiation was observed (p < 0.001). However, protein expression did not correlate with any known prognostic variables [4].

Kohlberger et al. found 21% HER-2/neu oncoprotein expression and according to their study, clinical stage, histologic stage, histologic grade and depth of invasion did not correlate with c-erbB-2 oncoprotein expression [20]. Therefore c-erbB-2 oncoprotein was observed in all clinical stages and it did not seem to be a late event in the natural history of EC. They also reported that c-erbB-2 oncoprotein expression was associated with poor overall survival (long-rank P-value 0.04) [20]. Khalifa et al. reported that c-erbB-2 overexpression was significantly associated with depth of myometrial invasion [22]. Coronado et al. found that prognostic value of the c-erbB-2 overexpression was higher in early stages than in advanced stages of disease. The authors commented that this might have been related to the higher levels of overexpression found in serous and G3 cancers [21].

We observed 18.1% positive staining with c-erbB-2 in our study; and there wasn't any expressive relation with c-erbB-2 and respectively menstrual status, age or depth of myometrial invasion. In our research, even though we determined approximately the same ratio of c-erbB-2 positivity in G1 and G3 tumors, we found out that the ratio of c-erbB-2 positivity was relatively high in G2 tumors. C-erbB-2 staining and grade did not show a statistically significant relation (p > 0.05). Most of the cases were in G2 and the statistical results might be related with the number of G2 cases and the insufficient number of G3 cases. We were able to make "staging" for 34 of the cases and most of them were at stage I. In our study, cerbB-2 positivity was determined in stage I and stage III tumors. There was only one case in stage II and there was not any c-erbB-2 expression. Even though we determined a relatively high proportion of c-erbB-2 expression in stage III tumors, there was not any statistically significant correlation because of the insufficient number of stage III cases.

In the study by Grushko et al. HER-2 gene amplification was detected in 44%. There was a significant association between the grade and HER-2 amplification among nonserous tumors. Neither overexpression nor amplification predicted overall survival after adjusting for treatment and performance status [7]. Backe et al. also showed that immunohistochemically detected expression of c-erbB-2 was not a clinical prognostic factor in endometrial cancer [17].

In our study there was not a statistically significant relation between survival and c-erb-B-2. This result might be due to the insufficient number of followed-up patients and the short time period.

There are studies revealing that c-erbB-2 is a poor prognostic factor or it has no relation with prognosis. Because of that the c-erbB-2 immunstaining is difficult to use as a predictor for prognosis [18]. It is well-known that anti c-erbB-2 antibodies have very variable sensitivity in formalin-fixed, paraffin-embedded tissue, and that other technical issues are also critical [23]. The variability in c-erbB-2 immunostaining results in the literature is probably related both to a different mix of patients in different series, and the wide variety of antibodies and staining conditions used. However the number of cases with c-erbB-2 overexpression is small, in both our series and in the other series in the literature, limited the precision of the results.

It is well known that cytosolic ERs and PRs can be identified in EC and a correlation between the contents of ER and PR within individual lesions has been demonstrated. Well differentiated tumors are more likely to be ER and PR positive than poorly differentiated neoplasms. So, the presence of ER and PR in EC is suggested to be inversely proportional to the nuclear grade and FIGO stage of the lesion [10]. In a study, ER and PR expression in EC was significantly associated with both well-differatiated and early-stage ECs which were positive for both ER and PR. (19). In the study by Bigsby et al. c-erbB-2 oncogene protein, epidermal growth factor receptor, ER, and PR were examined immunohistochemically in specimens of normal and neoplastic endometrium. There was an inverse relationship between overexpression of c-erbB-2 and PR in EC. On the other hand, the overexpression of c-erbB-2 in EC did not seem to be related to the loss of other differentiated characteristics [24].

Berchuck et al. in their study reported that high expression of c-erbB-2 was found in 27% of patients with metastatic disease compared with 4% of patients with disease confined to the uterus (p < 0.005). High cerbB-2 expression also was associated with absence of ER (p < 0.005) and with increased mortality from cancer [25]. Benevolo et al. reported that c-erbB-2 expression also influenced the patient outcome in the group with tumors lacking PRs (p = 0.004). In multivariate analysis PRs and c-erbB-2 overexpression emerged as independent prognostic factors [3].

In our study, in low grade tumors the number of ER and PR positive cases were relatively high. There was a statistically significant relation between PR and the grade. PR expression ratio decreased in high grade tumors. C-erbB-2 staining and ER did not show a statistically significant relation (p > 0.05). However, it is noteworthy that, in ER negative cases, c-erbB-2 expression was high. We found a statistically significant relation between c-erbB-2 and PR (p < 0.05). There was an inverse relationship between overexpression of c-erbB-2 and PR in EC. In PR negative cases, expression of cerbB-2 was statistically significantly high. On the other hand; there was not any statistically significant relation between ER-stage-survival and PR-stage-survival. Even though we couldn't find any relation between these variables, we thought that absence of PR might be related to the worse clinical process depending on literature. Moreover, the absence of PR and c-erbB-2 expression in EC, which is known as endocrine related neoplasm, could be a reliable parameter in the selection of specific hormonal treatment models.

## Conclusions

As a result, our study showed that c-erbB-2 positivity did not have a significant relation with menstrual status, histologic grade, myometrial invasion, lymph node status, stage, survival or ER status. It was found to be related with only PR. However we believe that these results provide additional evidence of the potential prognostic role of c-erbB-2, and it is worthwhile to investigate this marker with further prospective and controlled studies.

## Competing interests

The authors declare that they have no competing interests.

## Authors' contributions

AEG and SHK carried out the evaluations of immunohistochemistry results and drafted the manuscript. NK and CC participated in the design of the study and SS performed the statistical analysis. NOK and NOB participated in its design and coordination. All authors read and approved the final manuscript.
